# Aligning text mining and machine learning algorithms with best practices for study selection in systematic literature reviews

**DOI:** 10.1186/s13643-020-01520-5

**Published:** 2020-12-13

**Authors:** E. Popoff, M. Besada, J. P. Jansen, S. Cope, S. Kanters

**Affiliations:** 1 Precision HEOR, 1505 West 2nd Ave #300, Vancouver, British Columbia V6H3Y4 Canada; 2 Precision HEOR, Oakland, CA USA; 3 School of Population and Public Health, University of British Columbia, Vancouver, British Columbia Canada

**Keywords:** Study selection, Machine learning, Text mining, Systematic literature reviews, Methods, Updates, Classification, Reasons for exclusion, Downsampling

## Abstract

**Background:**

Despite existing research on text mining and machine learning for title and abstract screening, the role of machine learning within systematic literature reviews (SLRs) for health technology assessment (HTA) remains unclear given lack of extensive testing and of guidance from HTA agencies. We sought to address two knowledge gaps: to extend ML algorithms to provide a reason for exclusion—to align with current practices—and to determine optimal parameter settings for feature-set generation and ML algorithms.

**Methods:**

We used abstract and full-text selection data from five large SLRs (*n* = 3089 to 12,769 abstracts) across a variety of disease areas. Each SLR was split into training and test sets. We developed a multi-step algorithm to categorize each citation into the following categories: included; excluded for each PICOS criterion; or unclassified. We used a bag-of-words approach for feature-set generation and compared machine learning algorithms using support vector machines (SVMs), naïve Bayes (NB), and bagged classification and regression trees (CART) for classification. We also compared alternative training set strategies: using full data versus downsampling (i.e., reducing excludes to balance includes/excludes because machine learning algorithms perform better with balanced data), and using inclusion/exclusion decisions from abstract versus full-text screening. Performance comparisons were in terms of specificity, sensitivity, accuracy, and matching the reason for exclusion.

**Results:**

The best-fitting model (optimized sensitivity and specificity) was based on the SVM algorithm using training data based on full-text decisions, downsampling, and excluding words occurring fewer than five times. The sensitivity and specificity of this model ranged from 94 to 100%, and 54 to 89%, respectively, across the five SLRs. On average, 75% of excluded citations were excluded with a reason and 83% of these citations matched the reviewers’ original reason for exclusion. Sensitivity significantly improved when both downsampling and abstract decisions were used.

**Conclusions:**

ML algorithms can improve the efficiency of the SLR process and the proposed algorithms could reduce the workload of a second reviewer by identifying exclusions with a relevant PICOS reason, thus aligning with HTA guidance. Downsampling can be used to improve study selection, and improvements using full-text exclusions have implications for a learn-as-you-go approach.

**Supplementary Information:**

The online version contains supplementary material available at 10.1186/s13643-020-01520-5.

## Background

Systematic literature reviews (SLRs) are becoming more demanding given the ever-growing number of publications and the increasing breadth of research questions. In addition, health technology assessment (HTA) agencies, such as the National Institute for Health and Care Excellence (NICE), are expected to shorten the timeliness of technology appraisals to expedite access to clinically and cost-effective technologies [1]. Typically, guidelines for SLRs recommend that study selection is performed with a two-step process where potentially relevant studies are identified by screening title and abstracts followed by a review of the full-text reports of the potentially relevant studies not excluded during abstract screening. This process is typically conducted by two independent reviewers and their results are collated to reduce the amount of errors [2–4]. Reviewers need to keep a clear record of reasons for excluding studies based on eligibility criteria during full-text review, but it is also recommended to do so during the abstract screening phase [2–6]. For example, the Pharmaceutical Benefits Advisory Committee (PBAC) does provide an example PRISMA diagram that includes reasons for exclusion both at abstract and full-text review levels. Providing the reason for exclusion for both abstract and full-text screening helps the rigor of the review, facilitates assessing its quality, and can be valuable for updates. The most common methodological criticism in an SLR used for a technology appraisal submitted to NICE has been identified as the lack of transparency in the reviewer’s process for study selection [7]. However, recording reasons for exclusion at both the abstract and full-text review level is a time-consuming process.

Machine learning algorithms, a tool of artificial intelligence, are computational procedures that, among other things, use pattern recognition and inference by learning from previously categorized documents to predict the category a new document belongs to [8]. These algorithms are trained using example data in order to learn the patterns required to conduct the requisite classification. As these are quantitative procedures, it is necessary to generate a feature-set to convert information into an amenable format (i.e., from words to numerical data). In the context of an SLR, the title and abstract of a given study can be represented by a “bag-of-words,” which stores the words but ignores the grammar and word order while retaining the count of any repeated words. The choice of data included in this step, as well as the approach used to represent this information, may influence the classification results during the subsequent application of machine learning algorithms.

Machine learning algorithms have been suggested to reduce the burden on researchers during the study selection process of an SLR by reducing the number of abstracts that need to be screened or by replacing the need for a second human screener [8–10]. O’Mara-Eves et al. explored the evidence base related to the automation of screening and identified 44 studies published between 2006 and 2014 [10]. They concluded that reducing the number of citations needed to screen is the most common approach employed by developers (30 out of 44 papers), which was achieved by removing the more obvious studies not meeting the study selection criteria, yielding a workload reduction of between 10 and 90%. Alternatively, six papers advocated for the use of text mining to replace a second screener. In this approach, one reviewer screens all the records, while the machine acts as an independent “fact-check”. Additionally, seven papers used automation to increase the speed of screening, and 12 papers improved workflow through screening prioritization. Bekhuis et al. found this approach reduced workload for the second screener by 88% to 98% [11]. This approach may be more accepted by HTA agencies, given that one reviewer is retained for all citations, and a second reviewer is used for any challenging citations not excluded by the algorithm. The limited feasibility of dual screening has been recognized by the Patient-Centered Outcomes Research Institute (PCORI), who suggested that “fact-checking may be more sufficient” [12].

Machine learning methods proposed for study selection in SLRs have some limitations that may be important to address in order to support HTA submissions, which require a high level of transparency. Firstly, machine learning algorithms tend to be regarded as being “black boxes.” For the purpose of study selection, it is not sufficient to provide an include/exclude decision as typically provided by machine learning algorithms; there must be a reason for exclusion according to the PICOS (population, intervention, comparator, outcomes, and study design) framework [2, 4, 6]. Second, despite existing research on text mining and machine learning for title and abstract screening, the best algorithm remains unclear. Although the most commonly used algorithms are support vector machines (SVMs) [8, 13], some literature suggests that alternative options, such as naïve Bayes, may be more favorable [9]. Third, with respect to optimizing algorithms, a key critique of existing machine learning applications to study selection is that it seems parameter settings often rely on default settings; in other words, authors may not have systematically evaluated feature generation and classification algorithms in order to determine the impact and validity of such tools [13].

Our objective was to develop an algorithm for SLR abstract screening that excludes abstracts according to the specific reasons defined according to the PICOS framework. In doing so, we also compared factors pertaining to data preparation, training set settings, feature-set selection, and classification algorithm, in order to optimize study selection by maximizing sensitivity, followed by specificity.

## Methods

We used data from existing SLRs (further described below) to derive both training and test sets with which to apply machine learning algorithms for study selection at the abstract screening phase. We conducted simulations across a large variety of scenarios as summarized in Table 1 in order to gain insight into how a set of choices pertaining to the feature-set generation, and classification algorithms could be used to optimize identification of citations to exclude. Finally, the simulations were conducted using an overall process that allowed to keep track of reasons for exclusion. The full details are provided below.

**Table 1:** Datasets and model parameters considered across 870 simulations

Datasets (size)	Data scenarios	Downsampling	Word frequencies	Classification algorithms	Model metrics^**b**^
Psoriasis (4442)	Abstract screening	With	Removing words appearing < 5 times across all citations	SVM	ROC
Lung cancer (12,769)	Full-text screening	Without	Removing words appearing < 10 times across all citations	Naïve Bayes	Sensitivity
Liver cancer (8507)	Removing full-text excludes		Removing words appearing < 100 times across all citations	Bagged CART	
Melanoma (3089)			Removing words appearing < 500 times across all citations		
Obesity (5187)			Keeping top 50 words in terms of variable importance^a^		
			Keeping top 100 words in terms of variable importance^a^		
			Keeping top 500 words in terms of variable importance^a^		

^a^Not applicable to the SVM algorithm

^b^Not applicable to the bagged CART algorithm

### Data and construction of training and test sets

Given that we opted to use data from previously conducted SLRs to both construct training and test sets, the training set can be seen as being analogous to conducting an update of a previously performed SLR. The five datasets we used were from large existing SLRs that varied in disease areas: psoriasis (*n* = 4442), lung cancer (*n* = 12,769), liver cancer (*n* = 8507), melanoma (*n* = 3089), and obesity (*n* = 5187). Each of the SLRs was guided by pre-defined eligibility criteria, and screening decisions were verified through the involvement of two independent researchers in the study selection process. A brief summary of the SLRs is provided in Table 2. Data for each reference consisted of an abstract, a decision (include/exclude), and a reason for exclusion where applicable (every excluded abstract did contain a reason for exclusion). With respect to decisions and reasons for exclusion, three different approaches were considered: (1) abstract screening (i.e., all decisions based on abstract screening only); (2) full-text screening (i.e., references excluded at full-text were identified as excludes for the specified reason); (3) abstract screening for excludes and full-text screening for includes (modified full-text; i.e., removing references included at abstract-level but excluded at full-text screening). Full-text decisions are more informative and would be used if available. The motivation for approach #3 is to assess how much is lost when using abstract decisions to better understand its impact on learn-as-you-go algorithms.

**Table 2:** Summary of systematic literature reviews included

Datasets	Selection criteria		Abstract screening		Full-text screening
	Population	Study design	Abstracts screened, ***n***	Abstracts included, ***n*** (%)	Full-texts included, ***n*** (%)^
Psoriasis	Adults patients with moderate to severe psoriasis	RCTs and observational studies	4442	613 (13.8)	171 (27.9)
Lung cancer	Adult patients with advanced or metastatic lung cancer	RCTs	12,769	215 (1.7)	66 (30.7)
Liver cancer	Adult patients with unresectable liver cancer	RCTs and observational studies	8507	1141 (13.4)	294 (25.8)
Melanoma	Adult patients with unresectable stage III or IV melanoma	RCTs	3089	124 (4.0)	41 (33.1)
Obesity	Adult patients with BMI ≥ 25 kg/m^2^	RCTs with trial duration ≥ 12 months	5187	228 (4.4)	47 (20.6)

**Abbreviations**: *RCTs,* *randomized controlled trials*

^Percentage of included abstracts selected for full-text screening

All of the SLRs used were conducted in dual and all values used in the datasets were those agreed upon after reconciliation. Duplicated publications were identified and removed prior to proceeding. These data were split into training and test sets (75% training/25% test) via partitioned random sampling (include/exclude decision) with a constant random seed. This 75/25% split is for the purpose of these experiments to mimic an SLR update scenario and does not represent real-world practice. All parameter settings, chosen algorithms, and other model settings were defined a priori and were not influenced by examining the results on the test set.

### Feature generation

Feature generation refers to selecting the most relevant properties of the data such that these features provide the most value in the predictive model. In this context, we are attempting to find words or phrases (features) in an abstract that allow us to differentiate between the abstract being included or excluded as well as the reason for exclusion.

Feature generation was completed using a text mining bag-of-words approach. First, common English words, numbers, and punctuation were removed from all citations. Next, a word frequency matrix was created, and from this, a document-term matrix was formed. The frequency matrix counts the frequency of words within each document and the document-term matrix describes where words appear across the documents (here, citation abstracts). The latter has rows corresponding to each of the citations and columns corresponding to each term [14]. Additionally, we compared results when including or excluding rare occurring words. Rare words were defined as those appearing less than 5, 10, 100, or 500 times across the abstract text from all citations combined. Finally, zero-variance predictors (i.e., predictors with only one level that appear exclusively in either the training or test set) were removed from both the training and test set as these are useful to the model but can create numeric instability.

### Classification algorithms

A classification algorithm is a technique used to weigh the features of a dataset to create the optimal split of that data into two or more classes. In this context, we are classifying abstracts as being included or excluded (with a reason for exclusion).

Three machine learning classification algorithms were fit using each training set: (1) SVM; (2) naïve Bayes; (3) bagged CART. Each algorithm has its own set of tuning parameters, which rather than relying on the default settings of the tuning parameters, we considered varying values of the tuning parameters for each algorithm to ensure that differences between the algorithms were not biased by them. For SVM, the tuning parameter was cost (set to 0.25, 1, 2, 8, 32, 256). The cost describes the degree to which data points are penalized when they fall too close to the region separating the possible classification outcomes. The larger the cost, the more flexible the region is, where low costs lead to a smooth region of separation. A cost that is too high can lead to an extremely “bendy” region of separation, which in some cases may lead to overfitting. For the naïve Bayes model, the distribution type parameter was set to kernel or no kernel and the Laplace correction was set to 0, 0.5, or 1. Distribution type refers to whether or not a parametric normal distribution or a non-parametric distribution is used. Laplace correction refers to adjusting probabilities of rare occurring features (i.e., a probability equal to zero) to give them a small chance of occurring in order to avoid prediction probabilities of zero (given the multiplicative nature of the naïve Bayes model) [15].

When training these algorithms, we tested the effects of downsampling (also referred to as under sampling), which is the term used to describe the process of class balancing. This involves randomly removing a number of excludes (the larger class) from the data until the number of includes and excludes in the training set are equivalent. Within SLRs, it is often the case that there are many more excluded citations compared to included publications. This can cause major class imbalances when fitting classification algorithms to these data. Class imbalances can create complications in terms of training the algorithm, since many models will tend to overfit to the majority class which will have a negative impact on model performance [16]. In an attempt to rectify this, we reduced the number of excludes when conducting feature generation in order to balance the over-represented class (excludes) with the under-represented class (includes). Downsampling has been shown to work well in the literature with respect to increasing the sensitivity of the classifier in the under-represented class [17–19]. As a result, our approach also employed downsampling on a 1:1 basis and involved random sampling from the excludes to match the sample size of the includes.

An additional consideration that was required for working with the classification algorithms, was the choice of threshold to categorize a given citation as an include or exclude. The output provided by each algorithm is a probability of the citation being an include or exclude. As this was not the primary concern of this study, preliminary tests were used to identify a threshold. A threshold of 90% probability of exclusion was chosen because it maximized a model’s receiver operating characteristic (ROC) curve (closest point to the upper left corner).

Note that the tuning parameters were used in each model iteration and were not looped over separately. Moreover, both the ROC and sensitivity were used as performance metrics for these tuning parameters. Finally, in addition to using word frequency for feature-set generation, the above algorithms were fit using the top 50, 100, and 500 words based on variable importance using criteria specific to each individual algorithm (not applicable to SVM algorithm).

### Process for exclusion classification

**Fig. 1:**
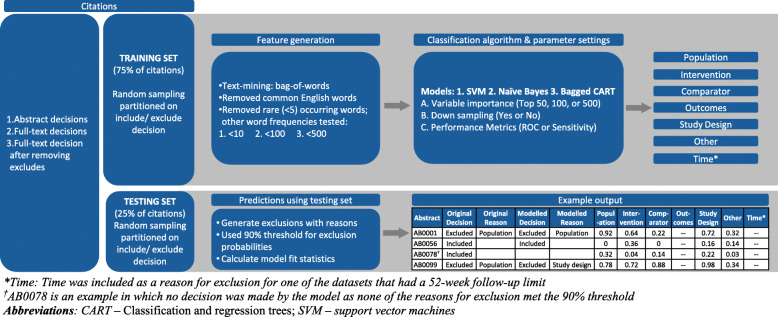
Complete study selection process to identify studies to exclude with reasons, including algorithm training and parameter setting

Figure 1 displays the overall process we developed for abstract screening classification. With this process, a collection of abstracts can be classified as excluded with a reason or undecided (to be reviewed by a human). The machine learning algorithms were first fit on overall include/exclude decision and then subsequently fit pairwise on include/specific exclusion reason (population, intervention, outcomes, study design, other, and time). Ten-fold cross-validation (CV) was performed on each model run. As tuning parameters and metrics differed between models, the CV-folds were randomized for each model. All these models were then used to predict the outcome of the test set citations and produced a result of either an include, exclude with reason, or unknown (left for human to screen). Exclusion reasons were selected from one of the following: study design, population, intervention, comparator, outcomes, other (i.e., study protocols, conference abstracts, language), time. When multiple reasons were applicable to a given citation, the exclusion reason was chosen based on a hierarchy in the order of the aforementioned reasons.

### Statistical analyses

The statistical analyses consisted of conducting simulations across the various scenarios to be compared. Table 1 highlights all datasets and model parameters considered. Altogether 870 scenarios were considered (420—naïve Bayes, 240—SVM, and 210—bagged CART). Model performance was assessed using sensitivity, specificity, run time, and percentage of correct exclusion reasons. In this context, we defined sensitivity as TP/(TP + FN), where TP (true positive) is a true included citation identified as an include or no decision and FN (false negative) is a true included citation identified as an exclude with a reason for exclusion. Specificity was defined as TN/(TN + FP), where TN (true negative) is a true excluded citation identified as an exclude with a reason for exclusion, and FP (false positive) is a true excluded citation identified as an include or identified as having no decision. These calculations were performed relative to the full-text decisions (i.e., if a citation was ultimately included or excluded in the final set of SLR citations).

Sensitivity and specificity were compared using a paired *t*-test in which each factor tested was held constant one at a time, while varying all other model characteristics (e.g., downsampling and performance metric). When selecting the best fitting model, we prioritized sensitivity given it is important not to incorrectly exclude any citations to be conservative in an SLR. If the opposite happens and a citation meant to be excluded is accidentally classified as an include, this causes minimal extra work and will likely be picked up by the human reviewer. After maximizing sensitivity, specificity was maximized as a secondary measurement. Exclusion reason percentage, exclusion reason matching percentage, and model run time were not considered heavily during the model selection process.

All simulations, as outlined in Table 1, were conducted in R (v3.4.4). Text mining and machine learning algorithms were obtained through the caret (v6.0-80) package via the following sub packages: klaR (v0.6-14), ipred (v0.9-6), and e1071 (v1.7-0). The R code is available in Additional file 1.

## Results

**Fig. 2:**
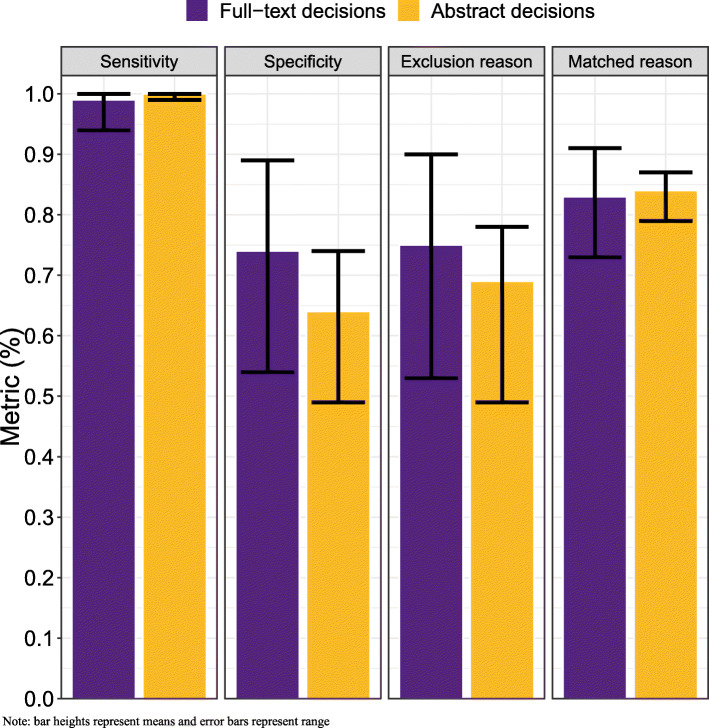
Comparing best fitting full-text decisions (SVM, frequency 5, ROC, downsampling) and abstract decision (SVM, frequency 5, sensitivity, downsampling) algorithm and settings

Overall, the best fitting algorithm and settings based on the model selection criteria was the SVM algorithm with the data set built using inclusion/exclusion decisions from the SLR’s full-text screening; feature-set generation using downsampling of exclusions; the removal of words occurring fewer than 5 times; and selecting an optimal cost using the ROC metric (note that no definitive cost was preferred over others between the five datasets). Statistics related to this combination of algorithm and settings can be found in Fig. 2 (labeled as “Full-text decisions”). The SVM algorithm using these settings had a sensitivity of 100% in all but two of the datasets and had an average sensitivity of 99% (range, 94 to 100%) and an average specificity of 74% (range, 54 to 89%) across the datasets. True included citations at full-text that were misclassified as excludes in the testing sets across datasets were as follows: psoriasis (2/33); lung cancer (0/17); liver cancer (1/76); melanoma (0/10); obesity (0/11). Table 3 presents the performance statistics across each dataset (means and standard deviations). Of the citations that were identified as excluded in the first stage of categorization, on average 75% (range: 53% to 90%) were excluded with a reason. In the second stage of categorization, the model correctly identified the reviewers’ original reason for exclusion, on average 83% (range, 73 to 91%) of the time.

**Table 3:** Model results for each dataset while varying all model characteristics

Datasets	Sensitivity mean (SD)	Specificity mean (SD)	Precision mean (SD)	Accuracy mean (SD)	Correct reason for exclusion^**a**^ mean (SD)
Psoriasis	84.97% (14.99%)	75.72% (15.84%)	19.82% (9.55%)	76.17% (14.63%)	88.66% (7.76%)
Lung cancer	77.01% (21.36%)	90.98% (9.05%)	10.94% (7.19%)	90.87% (8.88%)	93.78% (5.89%)
Liver cancer	84.23% (12.81%)	67.66% (18.69%)	19.38% (8.65%)	68.73% (16.87%)	82.58% (7.60%)
Melanoma	88.05% (22.11%)	87.05% (17.09%)	27.60% (21.71%)	87.07% (16.67%)	89.31% (7.84%)
Obesity	78.45% (23.95%)	84.80% (15.10%)	13.25% (10.25%)	84.71% (14.68%)	82.18% (16.02%)

Sensitivity = TP/(TP + FN), specificity = TN/(TN + FP), precision = TP/(TP + FP), and accuracy = (TP + TN)/(TP + FP + FN + TN); where TP (true positive) is a true included citation identified as an include or no decision, FN (false negative) is a true included citation identified as an exclude with a reason for exclusion, TN (true negative) is a true excluded citation identified as an exclude with a reason for exclusion, and FP (false positive) is a true excluded citation identified as an include or identified as having no decision

^**a**^Correct reason for exclusion was defined as the number of citations whose true reason for exclusion fell above the 90% threshold over the total number of citations with any reason for exclusion. Sensitivity, specificity, precision, and accuracy were calculated by holding each factor constant while averaging over all other model characteristics (e.g., downsampling and performance metric)

**Abbreviations**: *SD*, *standard deviation*

### Data generation

**Fig. 3:**
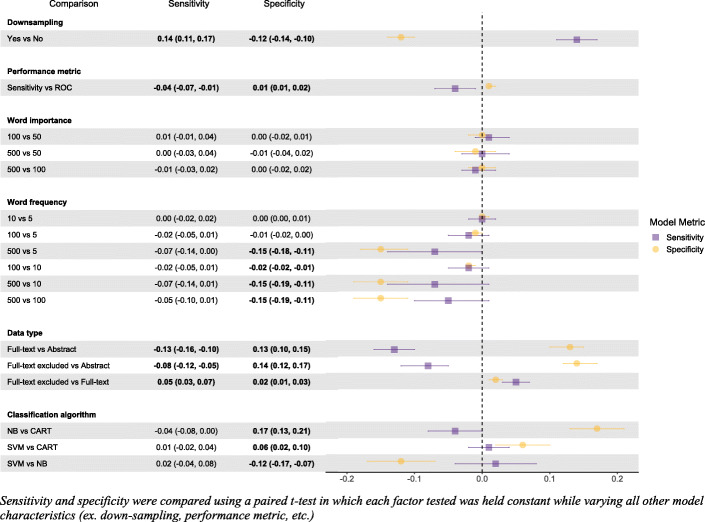
Comparative model results across all tested model characteristics

The inclusion/exclusion decisions used to create the training set impacted results and consequently slightly changed the choice of optimal algorithm. Specifically, if inclusion/exclusion were based on decisions from the SLR’s abstract screening only (e.g., suppose full-text decisions were not available), the best machine learning algorithm was again the SVM algorithm. Statistics related to this model can be found in Fig. 2 (labeled as “Abstract decisions”). Note that downsampling and removal of words occurring fewer than 5 times continued to be optimal, while selecting an optimal cost parameter for the SVM algorithm now used a sensitivity metric rather than a ROC metric. Comparisons of model performance in terms of sensitivity and specificity across all competing metrics are summarized in Fig. 3.

Averaged across all combinations, sensitivity was higher using abstract decisions than full-text decisions and modified full-text decisions. This makes sense given that abstract decisions are more inclusive and therefore will lead to more includes and less false negatives. In contrast, both full-text and modified full-text decisions led to higher specificity. Of note, modified full-text decisions improved both specificity and sensitivity relative to full-text decisions. All of the listed comparisons were statistically significant.

### Feature generation

Table 4 presents the performance statistics across each of the factors of interest. With respect to feature generation, on the basis of word frequency, keeping words that appeared at least 500 times performed much worse at lower thresholds, especially with respect to specificity. The thresholds did influence run times, but this carried very little weight in determining the best choice. Using words with a frequency of 10 or more words to build the feature-set led to the best specificity, while using words with a frequency of 5 or more words led to the best sensitivity.

**Table 4:** Model results for each factor while varying all other model characteristics

	Sensitivity mean (SD)	Specificity mean (SD)	Precision mean (SD)	Accuracy mean (SD)	Correct reason for exclusion^**a**^ mean (SD)
**Citation decisions**					
Abstract decisions	89.76% (10.74%)	70.50% (15.48%)	11.20% (4.53%)	71.43% (14.65%)	84.77% (6.12%)
Full-text decisions	76.49% (15.74%)	83.07% (13.02%)	20.11% (9.49%)	83.04% (12.27%)	85.63% (8.97%)
Modified full-text	81.37% (13.18%)	84.86% (12.07%)	24.23% (11.05%)	84.90% (11.42%)	87.52% (9.45%)
**Classification algorithms**					
CART	83.63% (16.56%)	70.35% (15.20%)	14.92% (7.91%)	71.00% (14.04%)	78.64% (7.72%)
NB	79.17% (11.41%)	88.45% (6.85%)	22.59% (10.14%)	88.30% (6.42%)	91.38% (4.41%)
SVM	87.20% (15.26%)	74.05% (16.39%)	15.43% (10.62%)	74.78% (15.67%)	84.76% (7.54%)
**Feature generation**					
Frequency = 5	85.67% (15.31%)	81.94% (13.09%)	20.07% (11.47%)	82.21% (12.23%)	87.40% (7.25%)
Frequency = 10	85.54% (14.29%)	82.13% (13.27%)	20.70% (12.26%)	82.39% (12.46%)	87.23% (7.29%)
Frequency = 100	83.52% (11.73%)	80.49% (13.38%)	18.19% (10.08%)	80.83% (12.59%)	86.82% (8.25%)
Frequency = 500	78.81% (19.22%)	67.38% (17.50%)	10.93% (6.62%)	68.20% (16.68%)	80.31% (6.91%)
Importance = 50	82.19% (9.54%)	85.44% (8.91%)	19.08% (7.17%)	85.51% (8.29%)	89.49% (6.29%)
Importance = 100	83.59% (9.19%)	85.10% (10.75%)	20.49% (8.27%)	85.22% (10.06%)	89.46% (7.47%)
Importance = 500	82.60% (10.75%)	84.73% (13.78%)	23.82% (11.18%)	84.84% (12.94%)	89.95% (7.45%)
**Performance metrics**					
ROC	83.90% (13.67%)	77.09% (15.32%)	17.09% (8.81%)	77.52% (14.44%)	84.02% (8.73%)
Sensitivity	80.07% (15.43%)	83.81% (13.34%)	21.10% (12.27%)	83.90% (12.59%)	89.53% (6.23%)
**Downsampling**					
Without downsampling	75.46% (15.87%)	85.55% (11.24%)	23.27% (10.93%)	85.45% (10.43%)	88.93% (5.90%)
With downsampling	89.62% (7.97%)	73.40% (15.79%)	13.76% (7.00%)	74.12% (15.06%)	83.01% (9.35%)

Sensitivity = TP/(TP + FN), specificity = TN/(TN + FP), precision = TP/(TP + FP), and accuracy = (TP + TN)/(TP + FP + FN + TN); where TP (true positive) is a true included citation identified as an include or no decision, FN (false negative) is a true included citation identified as an exclude with a reason for exclusion, TN (true negative) is a true excluded citation identified as an exclude with a reason for exclusion, and FP (false positive) is a true excluded citation identified as an include or identified as having no decision

^**a**^Correct reason for exclusion was defined as the number of citations whose true reason for exclusion fell above the 90% threshold over the total number of citations with any reason for exclusion. Sensitivity, specificity, precision, and accuracy were calculated by holding each factor constant while averaging over all other model characteristics (e.g., downsampling and performance metric)

**Abbreviations**: *SD,* *standard deviation*

There were no significant differences in either sensitivity or specificity when comparing models using the top 50, 100, and 500 words in terms of variable importance (Table 4, Fig. 3). This indicates that the classification algorithms are likely already removing the “noise” from their predictions, and sub-setting variables does not have much of an effect on model performance.

**Fig. 4:**
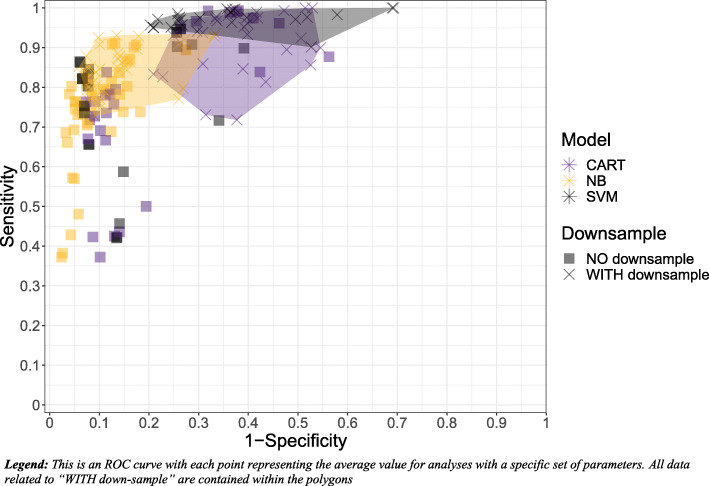
Sensitivity and specificity of model variations showcasing differences in classification algorithms and sampling

Downsampling played one of the most significant roles when examining model performance. As seen in Figs. 3 and 4, and Table 4, when downsampling was applied, this lead to a 14% higher sensitivity on average, while sacrificing −12% specificity and leading to decreased model run time. As we are most concerned with sensitivity and overall efficiency, incorporating downsampling was favored.

### Classification algorithm and performance metrics

**Fig. 5:**
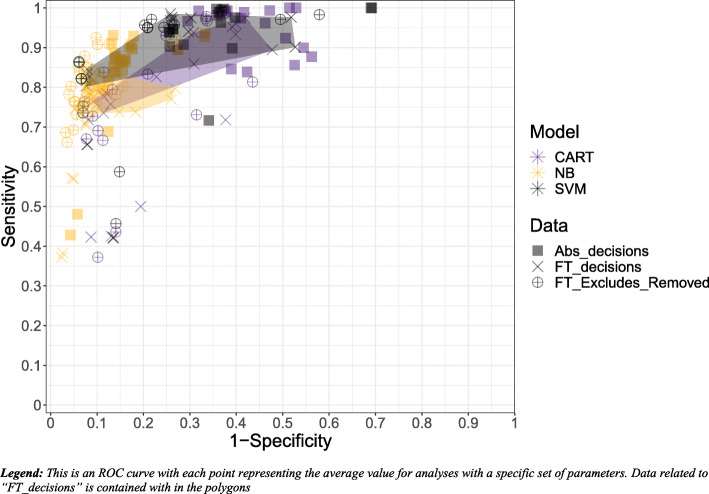
Sensitivity and specificity of model variations showcasing differences in classification algorithms and data decisions

Figures 3 and 5 illustrate the sensitivity and specificity across the model variations. Both CART and SVM had higher sensitivity than NB; however, these results were not statistically significant. With regards to specificity, NB was significantly better than both SVM and CART, while SVM was also significantly better than CART. Using sensitivity opposed to ROC as a performance metric ironically resulted in a significantly lower sensitivity and significantly higher specificity when compared with ROC.

### Overall process and time savings

The run times, using abstract decisions, ranged between 4.5 to 13.2 min for each dataset. The run times were faster using full-text decisions (3.0 to 6.8 min). Collectively, our entire dataset of 33,994 abstracts took a total of 31 min using abstract decisions. An SLR with 5000 abstracts can take an experienced reviewer between 60 to 85 h to complete abstract screening (approximately 500-800 abstracts per working day, which we assume to be 8 h) [8, 10], compared with 5 min for the algorithm. In any scenario, our process will take minutes to save hours of work.

## Discussion

Our study demonstrated a process that can be used to identify studies to exclude from an evidence base using machine learning and with the requisite transparency, namely, with a reason for exclusion. Moreover, our analyses provided new insights to optimize the process with the aim of maximizing sensitivity (i.e., to avoid falsely excluding citations that should be included). Taken together, text mining and machine learning can greatly increase the efficiency of the SLR process and could save many hours of manual effort. While choice of machine learning algorithm settings did influence results, data construction and feature generation had a greater impact. Among these, downsampling resulted in significantly improved sensitivity and run time (while sacrificing specificity), and the removal of excludes identified through full-text screening (modified full-text) resulted in significantly improved sensitivity, specificity, and reduced run times when compared to full-text decisions alone.

Despite existing research, uptake of machine learning algorithms to assist with SLRs remains low. One contributing factor may be that machine learning may be perceived as being “black-box”. In other words, it may be difficult to understand which inputs contribute to the classification of a given citation. This was a major point of contention during a panel discussion on the use of artificial intelligence within health economics and outcomes research at the ISPOR conference [20]. The methods evaluated in this paper increase the transparency of machine learning in the context of SLRs by providing a reason for exclusion which is aligned with the eligibility criteria described in the PICOS framework. Not only is this a standard in SLR procedures, but it is also a requirement for HTAs as it contributes to the transparency of the selection process within an SLR.

Given that not all citations can be excluded with a reason to guarantee a 100% sensitivity, we propose that the use of machine learning in combination with human screeners. One screener to screen all abstracts as would usually be done and the other to review the remaining abstracts that cannot be classified by the algorithm—a sort of cyborg reviewer. Another practical consideration relates to what scenarios this can be used for. For sake of simplicity, we focused on an SLR update, which represents the easiest scenario under which to construct a training set. Nonetheless, the algorithm is independent from the manner in which the training set is obtained and therefore could be used in other scenarios as well. Our study did not focus on the challenge of constructing a training set. As the cut point for an SLR is arbitrary, additional research is needed to quantify what constitutes a sufficient training set.

Finally, care should be taken in applying this algorithm to more complex situations. We found success using the algorithms to select RCT and observational designs, as well as to adult populations with a specific disease. However, further testing will be needed for more specific situations (e.g., patients with a specific line of treatment in oncology). For example, in the obesity data set, we re-categorized the trials excluded for short follow-up as exclusion for other reasons as it initially was leading to poor results.

In addition to allowing exclusions with reasons, three other key findings within our study are as follows: (1) the 10% decrease in specificity when using abstract-level decisions (with equivalent sensitivity) when using the best fitting models (Fig. 2); (2) the benefits of modified full-text decisions when compared to full-text decisions alone; and (3) the benefits of using downsampling. The improved specificity from using includes defined during full-text screening has implications for the “learn-as-you-go” approach that has been suggested by many, given that such a method is limited to abstract screening decisions only [10, 21]. Naturally, full-text screening decisions have the luxury of disaggregating true includes from those that reviewers have simply pushed forward from abstract to full-text screening for further investigation. Nonetheless, our study provides further insights into how much of a difference this makes. Specifically, among combinations leading to high sensitivity, using abstract-level decisions leads to approximately 10% lower specificity. Moreover, our hypothesis regarding the use of modified full-text decisions did prove to be correct. Namely, by removing exclusions from citations where the decision was almost surely made outside of the abstract (i.e., data and construction of training and test sets option #3; modified full-text), the algorithms improved with respect to using full-text for decision-making. This form of data generation improved both sensitivity and specificity.

The improved sensitivity and run time resulting from downsampling were somewhat counter-intuitive because in most contexts adding more data is helpful (e.g., more data leads to better estimates). In the context of machine learning, it is well known that these algorithms work best when the classes are well balanced [10]. As our study showed, in this context balance trumps sample size because reducing data led to improved analytical outcomes. Downsampling had an important impact on all three classification algorithms that we tested, but the impact was strongest in the SVM algorithm.

Our research complements research conducted by others. Garcia et al. tested various machine learning algorithms, such as SVM, NB, k-Nearest neighbors, and Rocchio, based on different parts of the articles (title, abstract, or both) [8]. Similar to our results, Garcia and colleagues concluded that SVM is superior to the other classifiers. The authors also identified that using only article titles provided comparable results to those when adding article abstracts. Frunza and colleagues used naïve Bayes and tested three feature selection methods (Chi2, InfoGain, and Bi-Normal Separation) and three representation techniques (bag-of-words, unified medical language system, and a combination of both). Using bag-of-words, Frunza et al. reported a sensitivity of 65.3% [9]. Our study suggested that NB resulted in a sensitivity of 79.17%.

There are some limitations to this study. Three classification algorithms were selected in this study based on the current literature; however, there are a multitude of other algorithms that have yet to be explored and tested. Similarly, with respect to feature generation, our study focused on a few factors, but there are many others that could also be explored. Focus on these particular factors was simply a result of conducting a feasibly sized study. The training sets were designed to mimic an SLR update and this may seem like a very narrow area of application; however, lessons learned through our study are not restricted by the manner in which our training sets were obtained and can be generalized to other applications. On the other hand, the training sets were quite large and it is unclear how smaller training sets would compare in terms of the measurements we used (e.g., specificity). A limitation to the factors identified as the preferred strategy may also be limited with respect to very large datasets, as the memory and run time required may be significantly impacted. Observed run times with models set to the most computational intensive settings varied from about 16 min for the smallest dataset, to 52 min in the largest dataset. Although this can be addressed using downsampling, other algorithms and techniques may be worth exploring to identify opportunities to increase efficiency.

In conclusion, in order to align with HTA requirements, machine learning algorithms for SLRs focused on study exclusions that should be accompanied with a PICOS reason. There are important steps in data preparation and feature-set generation that can have a very meaningful impact on study selection results. Most notably downsampling and using includes from the full-text stage can both improve sensitivity and specificity.

## Supplementary Information

**Additional file 1:.** Machine Learning Functions. Code used to apply the machine learning algorithms

## Data Availability

The datasets used and/or analyzed during the current study are available from the corresponding author on reasonable request.

## Abbreviations

CART: Classification and regression trees
CV: Cross-validation
FN: False negative
FP: False positive
HTA: Health technology assessment
NB: Naïve Bayes
ROC: Receiver operating characteristic
SD: Standard deviation
SLR: Systematic literature review
SVM: Support vector machine
TN: True negative
TP: True positive
